# *Suaeda glauca* Attenuates Liver Fibrosis in Mice by Inhibiting TGFβ1-Smad2/3 Signaling in Hepatic Stellate Cells

**DOI:** 10.3390/nu15173740

**Published:** 2023-08-26

**Authors:** You-Jung Hong, Gil-Hwan Kim, Yongdo Park, Hye-Jin Jo, Min-Woo Nam, Dong-Gu Kim, Hwangeui Cho, Hyun-Joo Shim, Jong-Sik Jin, Hyunsoo Rho, Chang-Yeob Han

**Affiliations:** 1 Institute of New Drug Development, School of Pharmacy, Jeonbuk National University, Jeonju 54896, Jeonbuk, Republic of Korea; 2 LED Agri-Bio Fusion Technology Research Center, Jeonbuk National University, Iksan 54596, Jeonbuk, Republic of Korea; 3 Department of Oriental Medicine Resources, Jeonbuk National University, Iksan 54596, Jeonbuk, Republic of Korea

**Keywords:** *Suaeda glauca* extract, smart farming system, liver fibrosis, hepatic stellate cells, TGFβ1

## Abstract

Chronic liver injury due to various hepatotoxic stimuli commonly leads to fibrosis, which is a crucial factor contributing to liver disease-related mortality. Despite the potential benefits of *Suaeda glauca* (*S. glauca*) as a natural product, its biological and therapeutic effects are barely known. This study investigated the effects of *S. glauca* extract (SGE), obtained from a smart farming system utilizing LED lamps, on the activation of hepatic stellate cells (HSCs) and the development of liver fibrosis. C57BL/6 mice received oral administration of either vehicle or SGE (30 or 100 mg/kg) during CCl_4_ treatment for 6 weeks. The supplementation of SGE significantly reduced liver fibrosis induced by CCl_4_ in mice as evidenced by histological changes and a decrease in collagen accumulation. SGE treatment also led to a reduction in markers of HSC activation and inflammation as well as an improvement in blood biochemical parameters. Furthermore, SGE administration diminished fibrotic responses following acute liver injury. Mechanistically, SGE treatment prevented HSC activation and inhibited the phosphorylation and nuclear translocation of Smad2/3, which are induced by transforming growth factor (TGF)-β1 in HSCs. Our findings indicate that SGE exhibits anti-fibrotic effects by inhibiting TGFβ1-Smad2/3 signaling in HSCs.

## 1. Introduction

Chronic liver injury and inflammation contribute to the development of fibrosis, which can be triggered by diverse factors, including hepatitis viruses, alcohol, hepatotoxic drugs, and metabolic conditions like obesity and diabetes [1]. If liver fibrosis remains unresolved, it can progress to cirrhosis, ultimately leading to hepatocellular carcinoma or hepatic failure, which is one of the key causes of morbidity and mortality globally [1,2,3]. Emerging evidence suggests that the pathological states of liver fibrosis can be reversible in both experimental and clinical situations [1,2]. However, there are currently no approved drugs that effectively target fibrosis. Therefore, it is crucial to search for novel candidate compounds and substances that possess anti-fibrotic properties and to understand the cellular and molecular mechanisms underlying their anti-fibrotic effects.

The formation of fibrous scars is the main characteristic of liver fibrosis due to the deposition of extracellular matrix (ECM) proteins primarily produced by hepatic stellate cells (HSCs) [1,2,4]. Under normal physiological conditions, quiescent HSCs are a major storage site for vitamin A. However, in pathological states caused by liver injury, HSCs lose their retinols and undergo activation, transdifferentiating into the activated HSCs [4]. The activated HSCs act as a myofibroblast, which drives the fibrotic responses and contributes to the development of liver fibrosis [4,5]. Thus, the inhibition of HSC activation has emerged as a promising approach for the treatment of liver fibrosis.

Natural products have been considered attractive candidates for the development of therapeutic agents in a wide range of human diseases. They offer potential resources for the treatment and/or prevention of liver fibrosis [6]. Among these natural products, *Suaeda glauca* (*S. glauca*) is a salt-tolerant annual halophyte from the Amaranthaceae family that is commonly found in inland saline soil and salt marshes along the coasts of South Korea, China, Mongolia, Siberia, and Japan [7]. Despite its wide distribution, there is limited information available regarding the therapeutic potential of *S. glauca* compared with other well-known natural products. One of its isoflavone constituents, Suaeglaucin B, has been reported to exhibit antioxidant activity [8]. Additionally, phenolic compounds derived from *S. glauca* have shown cytoprotective effects on a human hepatocyte cell line [9]. Nevertheless, the potential impact of *S. glauca* on liver fibrosis, particularly in relation to HSC activation, has not been investigated yet.

In the present study, we obtained *S. glauca* extract (SGE) from plants cultivated in an indoor smart farm facility using LED grow lights and employed in vivo animal and in vitro cell models to investigate the anti-fibrotic effects of SGE. Our findings revealed that treatment with SGE effectively inhibits liver fibrogenesis in mice and HSC activation. Mechanistically, these effects are attributed to the suppression of transforming growth factor-beta 1 (TGFβ1)-mediated Smad2/3 signaling in HSCs.

## 2. Materials and Methods

### 2.1. Materials 

Antibodies against p-Smad2 (#18338), p-Smad3 (#9520), Smad2/3 (#8685), c-Jun N-terminal kinase (JNK) (#9252), p-JNK (#9251), phosphorylated extracellular signal-regulated kinase (p-ERK) (#4376), and glyceraldehyde-3-phosphate dehydrogenase (GAPDH) (#2118) were obtained from Cell Signaling Technology (Danvers, MA, USA). Anti-ERK antibody (#AF1576) and the recombinant protein of TGFβ1 were acquired from R&D Systems (Minneapolis, MN, USA). Antibodies against proliferating cell nuclear antigen (PCNA) and α-Tubulin were provided by Bioworld Technology (Bloomington, MN, USA) and Santa Cruz Biotechnology (Dallas, TX, USA), respectively. Horseradish peroxidase-linked anti-rabbit (ADI-SAB-300-J) and anti-mouse (ADI-SAB-100-J) IgGs were provided by Enzo Life Sciences (Farmingdale, NY, USA). Antibodies against alpha-smooth muscle actin (α-SMA) (#A5228) and β-Actin (#A5441), as well as other reagents, were purchased from Sigma (St. Louis, MO, USA). 

### 2.2. Cultivation of S. glauca in a Smart Farming System 

*S. glauca* were collected and identified by Dr. Suk-Kyu Kim (Halopharm Co., Iksan, Republic of Korea) in the coastal area of Taean-gun, Republic of Korea. The seeds were grown for 45 days in a plant factory located at the LED Agri-bio Fusion Technology Research Center (Jeonbuk National University, Iksan, Republic of Korea). The room temperature for growth was maintained at 21 ± 1 °C using air conditioning and circulation fans. The relative humidity was kept at 60 ± 5% throughout the farming period. The artificial light source LED conditions (red:blue = 6:4 ratio) were set to 150 mmol/m^2^/s at 20 cm with a 16 h light/8 h dark cycle. The pH and electrical conductivity of the nutrient solution were kept within a range between 6.0 ± 0.5 and 2.2 ± 0.2 ms∙cm^−1^, respectively. An environmental control system was utilized to monitor all of these conditions.

### 2.3. Preparation of SGE

The leaves of *S. glauca* were washed and dried overnight in an oven at 60 °C. The dehydrated leaves were minced and extracted for 2 h in hot water (100 °C) using a reflux condenser with a solid to liquid ratio of 1:10 (*w*/*v*). The extract was filtered through Whatman filter paper No.1, lyophilized (batch method), and then stored at 4 °C before use. The percentage yield of the dried extract was 33.27% (*w*/*w*).

### 2.4. Animal Treatments

Animal experiments were performed following guidelines of the Institutional Animal Care and Use Committee at Jeonbuk National University. Seven-week-old male C57BL/6 mice were obtained from Samtako (Osan, Republic of Korea) and then accommodated in cages under standard conditions (20 ± 2 °C, 50–60% humidity, 12 h light–dark cycle). For the liver fibrosis model, the mice were injected intraperitoneally (*i.p.*) with vehicle (mineral oil) or carbon tetrachloride (CCl_4_) (0.6 mL/kg) twice a week for a total of six weeks. Starting from the second week, the CCl_4_-treated mice were divided randomly into three groups and were treated with vehicle (phosphate-buffered saline, PBS) or SGE (30 or 100 mg/kg) via oral gavage for the last five weeks. For the acute liver injury model, mice were orally administered SGE (100 mg/kg) four times every 24 h. One hour after the last SGE administration, the mice were subjected to a single injection of CCl_4_ (0.6 mL/kg, *i.p.*) and were sacrificed after 24 h.

### 2.5. Histology and Sirius Red Staining 

Paraffin-embedded liver tissue sections with a thickness of 5 μm were processed for hematoxylin and eosin (H&E) staining for light microscopy examination. For histological visualization of the collagen, a Picrosirius Red stain kit obtained from Abcam (Cambridge, MA, USA) was utilized. The experimental steps were conducted following the manufacturer’s protocol. Randomly selected areas were captured in a consistent and unbiased manner using a light microscope (Olympus, Tokyo, Japan). The entire slides were thoroughly examined to ensure staining quality, and a representative image was selected from multiple pictures. Staining intensities were assessed through the utilization of ImageJ 1.54d software.

### 2.6. Biochemical Analysis

The serum levels of alanine aminotransferase (ALT) and aspartate aminotransferase (AST) activity were quantified using commercially available kits (#K753 and #K752) following the manufacturer’s instruction (Biovision, Milpitas, CA, USA).

### 2.7. Western Blots 

Tissue or cell lysates were prepared by RIPA buffer on ice, and supernatants were added using sodium dodecyl sulfate (SDS) sample dilution buffer. The target proteins within the lysates were separated using polyacrylamide gels and subsequently transferred onto membranes made of nitrocellulose or polyvinylidene difluoride (PVDF). The blots were visualized using an enhanced chemiluminescence (ECL) system (Millipore, Billerica, MA, USA), and digital images of the bands were captured using an imaging system FUSION SOLO 6S (Vilber, Collégien, France). The relative band intensities were measured using ImageJ 1.54d software.

### 2.8. RNA Isolation and Quantitative Real-Time PCR Assays 

Total RNA was isolated using RNAiso Plus (#9109, Takara Bio, Shiga, Japan), and 1 μg of the obtained total RNA was subjected to reverse transcription to generate cDNA. Quantitative reverse transcription polymerase chain reaction (qRT-PCR) was conducted using a TOPreal qPCR 2X PreMIX (#RT500M, Enzynomics, Daejeon, Republic of Korea) and an ABI QuantStudio instrument (Applied Biosystems, Thermo Fisher Scientific, Waltham, MA, USA) according to the manufacturer’s instructions. The accuracy of each PCR amplicon was confirmed by obtaining a melting curve. The relative levels of each mRNA were normalized to glyceraldehyde-3-phosphate dehydrogenase (GAPDH) and calculated using the 2^−∆∆Ct^ method. The primer sequences utilized for qRT-PCR can be found in Table S1.

### 2.9. Cell Culture

The LX-2 (an immortalized cell line of human HSCs) was generously provided by Dr. S.L. Friedman (Icahn School of Medicine at Mount Sinai, New York, NY, USA). Primary HSCs were isolated from mouse liver as previously described [10]. The cells were cultured in Dulbecco’s modified Eagle’s medium (DMEM) containing 10% fetal bovine serum, 100 U/mL penicillin, and 100 μg/mL streptomycin at 37 °C in a humidified atmosphere with 5% CO_2_. The AML12 (a non-transformed murine hepatocyte cell line) was obtained from American Type Culture Collection (ATCC) (Rockville, MD, USA). The cells were cultured in the DMEM/F-12 comprising 10% FBS, 1% insulin–transferrin–selenium X (ITSX), dexamethasone (40 ng/mL), 100 units/mL penicillin, and 100 μg/mL streptomycin.

### 2.10. Immunofluorescence Staining

Mouse primary HSCs and LX-2 cells were seeded on a coverslip. The cells were then fixed with 4% paraformaldehyde and then permeabilized with 0.1% Triton X-100, followed by incubation with a blocking buffer. After blocking, primary antibodies directed against the proteins of interest were incubated overnight at 4 °C. Subsequently, AlexaFluor 488-conjugated goat anti-mouse IgG and AlexaFluor 594-conjugated goat anti-rabbit IgG secondary antibodies were incubated at room temperature. Following the incubation period, the samples were covered with a mounting solution and visualized using a Zeiss LSM 980 confocal microscope (Zeiss, Oberkochen, Germany).

### 2.11. MTT Assays

LX-2 or AML12 cells were plated in a 6-well plate to assess cell survival. After treatment with SGE for 24 h [or after exposure to CCl_4_ (5 mM, 24 h) following pre-treatment with SGE], viable cells were stained with 0.25 μg/mL of 3-(4,5-dimethylthiazol-2-yl)-2,5-diphenyl-tetrazolium bromide (MTT) (#M6494, ThermoFisher Scientific, Waltham, MA, USA) for 4 h. Subsequently, the media was aspirated, and the formazan crystals generated in the wells were dissolved by introducing dimethylsulfoxide. Absorbance readings were taken at 540 nm using a Multiskan SkyHigh ELISA microplate reader (ThermoFisher Scientific, Waltham, MA, USA). Cell viability was calculated relative to the untreated control (i.e., viability (%) = 100 × (absorbance of treated sample)/(absorbance of control)).

### 2.12. Migration Assay

Mouse primary HSCs were seeded in ibidi Culture-Insert 2 Well (ibidi, Grafelfing, Germany) and cultured for 3 days in the media containing SGE (100 μg/mL or 300 μg/mL). Then, the gasket was removed, and the cells were additionally incubated. The wound closure was evaluated using EVOS M7000 microscope over a 72 h period, and the width of the leading edges at the end point was subtracted from the width of the cell free gaps just after the gasket removal. The relative wound closure was calculated by image J (i.e., relative wound closure = (0 h cell free gaps − 72 h cell free gaps)/0 h cell free gaps).

### 2.13. Reporter Gene Assay

LX-2 cells were plated in a 6-well plate and were transfected the following day with the plasmid containing the luciferase gene under the control of Smad binding element (SBE). Subsequently, the media were changed to the serum-free media, after which the cells were exposed to TGFβ1 along with SGE. Luciferase activities were measured using a dual-luciferase reporter assay system (Promega, Madison, WI, USA). The activities were normalized to the Renilla luciferase control and were expressed as relative luciferase activities.

### 2.14. Statistical Analysis 

The data were presented as the mean ± standard error of the mean (SEM). Statistical significance was determined using the Student’s *t*-test or one-way analysis of variance (ANOVA) followed by Tukey’s post-hoc multiple comparison tests. Differences were considered significant at *p* < 0.05 or *p* < 0.01. Statistical analyses were conducted using GraphPad Prism 9.0 software (San Diego, CA, USA).

## 3. Results

### 3.1. SGE Treatment Effectively Inhibits CCl_4_-Induced Liver Fibrosis in Mice

**Figure 1:**
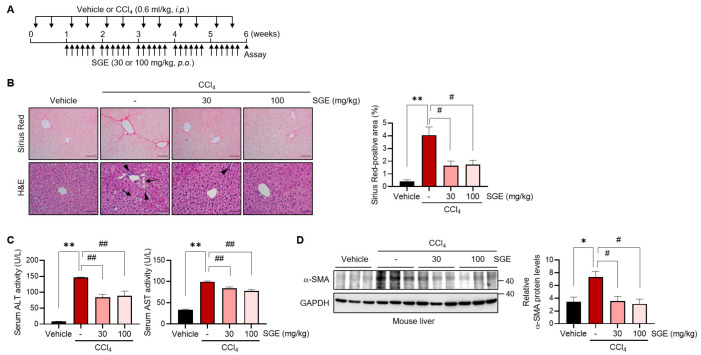
The effect of SGE on CCl_4_-induced liver fibrosis in mice. (**A**) A schematic diagram depicting the experimental setup for investigating the anti-fibrotic effect of SGE. (**B**) Sirius Red staining and H&E staining in the liver of mice (left) (scale bar: 20 μm). Mice were subjected to CCl_4_ treatment in the presence or absence of SGE, as shown in panel A. Arrows and arrowheads mark hepatocyte damage and macrophage infiltration, respectively. The area of Sirius Red positive staining was quantified (right) (*n* = 3 for each group). (**C**) ALT and AST activities were measured in the serum of mice (*n* = 6–8 for each group). (**D**) Immunoblotting for α-SMA in the liver of mice (left). The relative protein levels of α-SMA were quantified and normalized to those of GAPDH (right) (*n* = 3 for each group). The data are presented as mean ± SEM. The statistical significance of the differences between the groups was determined as follows: * *p* < 0.05 or ** *p* < 0.01 compared with the vehicle group and # *p* < 0.05 or ## *p* < 0.01 compared with the CCl_4_ alone group.

To assess the anti-fibrotic effect of SGE, we utilized the well-established CCl_4_-induced liver fibrosis animal model. C57BL/6 mice were injected with CCl_4_ for 6 weeks, and SGE was orally administered at either 30 or 100 mg/kg for the last 5 weeks of the injection (Figure 1A). The administration of SGE did not affect the body weight of mice treated with CCl_4_ (Figure S1). CCl_4_ treatment successfully induced hepatic fibrosis, as confirmed by Sirius Red staining, while the accumulation of collagen was significantly inhibited by SGE supplementation at both 30 and 100 mg/kg (Figure 1B). Histological changes assessed by H&E staining also demonstrated the protective effects of SGE on hepatocyte damage and macrophage infiltration (Figure 1B). Furthermore, SGE administration led to a significant decrease in ALT and AST activities in blood biochemical analyses, indicative of improved liver function (Figure 1C). SGE supplementation significantly reduced the protein expression of α-SMA, a marker of HSC activation induced by CCl_4_ (Figure 1D). Overall, these results demonstrate that SGE treatment inhibits CCl_4_-induced liver fibrosis.

### 3.2. SGE Treatment Prevents the Early Fibrogenic Response and Suppresses Inflammatory Genes in Mice

**Figure 2:**
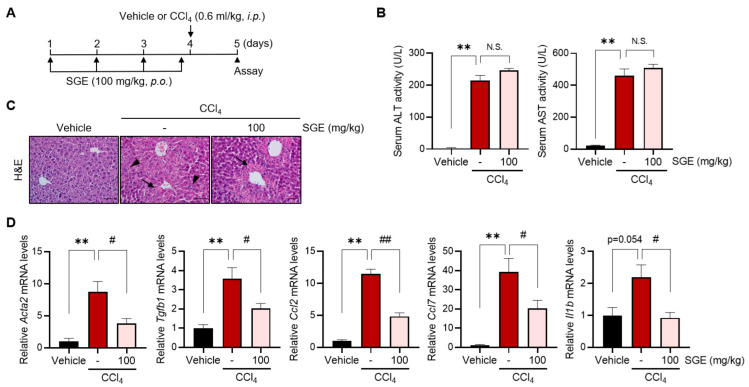
The effect of SGE on fibrogenic and inflammatory responses upon acute liver injury. (**A**) A schematic diagram depicting the experimental setup to evaluate the impact of SGE on CCl_4_-induced acute liver damage. (**B**) ALT and AST activities were measured in the serum of mice (*n* = 4–6 for each group). (**C**) H&E staining in the liver of mice (scale bar: 20 μm). Arrows and arrowheads mark hepatocyte damage and macrophage infiltration, respectively. (**D**) qRT-PCR assay for hepatic fibrogenic and inflammatory genes. Mice were treated according to the experimental protocol illustrated in panel A (*n* = 4–6 for each group). The data are presented as mean ± SEM. The statistical significance of the differences between the groups was determined as follows: ** *p* < 0.01 compared with the vehicle group and # *p* < 0.05 or ## *p* < 0.01 compared with the CCl_4_ alone group. N.S., not significant.

Based on the observed inhibition of liver fibrosis by SGE, we proceeded to examine whether SGE supplementation can also inhibit early fibrogenic responses following acute hepatotoxic challenges. Mice were administered SGE prior to a single injection of CCl_4_ (Figure 2A). Interestingly, the administration of SGE did not alleviate serum ALT and AST activities in response to acute CCl_4_ challenge (Figure 2B), similar to the results observed in H&E staining (Figure 2C). In line with our in vivo results, we also found that SGE treatment had no significant influence on the viability of AML12 cells, a hepatocyte cell line, both under basal conditions and in the context of CCl_4_-induced hepatotoxicity (Figure S2A,B). These results suggest that the anti-fibrotic effect of SGE in chronic liver injury may not be attributed to its early hepatoprotective effects. Next, we observed that the injection of CCl_4_ led to increased expression levels of fibrogenic genes, such as *Acta2* (*α-SMA*) and *Tgfb1*, as well as inflammatory genes, including *Ccl2*, *Ccl7*, and *Il1b*, in the liver of mice, whereas these elevations in gene expression were significantly attenuated by SGE supplementation (Figure 2C). Collectively, these findings suggest the potential of SGE treatment to suppress the hepatic fibrogenesis process by inhibiting HSCs.

### 3.3. SGE Treatment Effectively Impedes HSC Activation 

**Figure 3:**
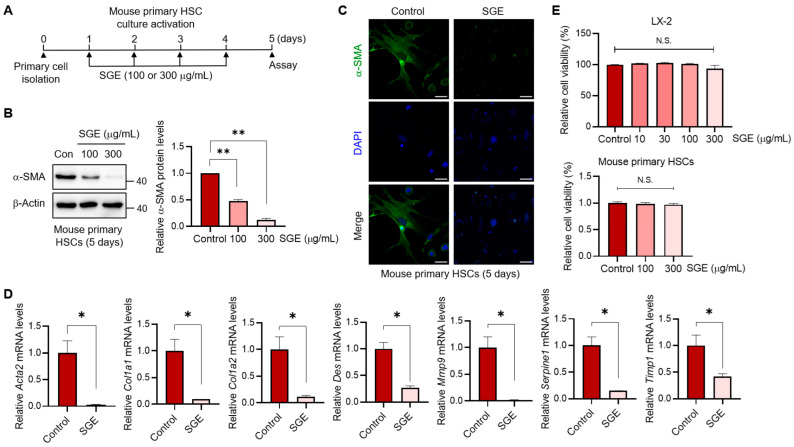
The effect of SGE on the fibrogenesis of HSCs. (**A**) Schematic diagram illustrating the experimental design for monitoring the effect of SGE on HSC activation. (**B**) Immunoblotting for α-SMA in mouse primary HSCs (left). Activated HSCs (5 days) were treated with SGE during the culture activation period, as depicted in panel A. The relative protein levels of α-SMA were quantified and normalized to those of β-Actin (right) (*n* = 3). (**C**) Immunofluorescence staining for α-SMA in activated HSCs (5 days) (scale bar: 20 μm). (**D**) qRT-PCR assay for fibrogenic genes in activated HSCs (5 days). Like panel A, the cells were treated with SGE (300 μg/mL). (**E**) MTT assay for cell viability in HSCs. Both LX-2 cells and mouse primary HSCs were treated with various concentrations of SGE as indicated for 24 h. The data are presented as mean ± SEM. The statistical significance of the differences between the groups was determined as follows: * *p* < 0.05 or ** *p* < 0.01 compared with the control group. N.S., not significant.

Given the critical role of HSC activation in the development and progression of liver fibrosis [4,11], we next explored whether the beneficial effect of SGE on liver fibrogenesis is indeed attributed to the inhibition of HSC activation. Primary HSCs isolated from mouse liver were treated with SGE at concentrations of 100 or 300 μg/mL during the culture activation for 5 days (Figure 3A). SGE treatment significantly and progressively reduced the expression levels of α-SMA in activated mouse primary HSCs (Figure 3B), which was further confirmed by immunofluorescence staining (Figure 3C). Moreover, SGE treatment suppressed the mRNA levels of fibrogenic genes in the HSCs (Figure 3D). Notably, the inhibition of fibrogenic gene expression in HSCs was not due to the cytotoxicity of SGE (Figure 3E).

**Figure 4:**
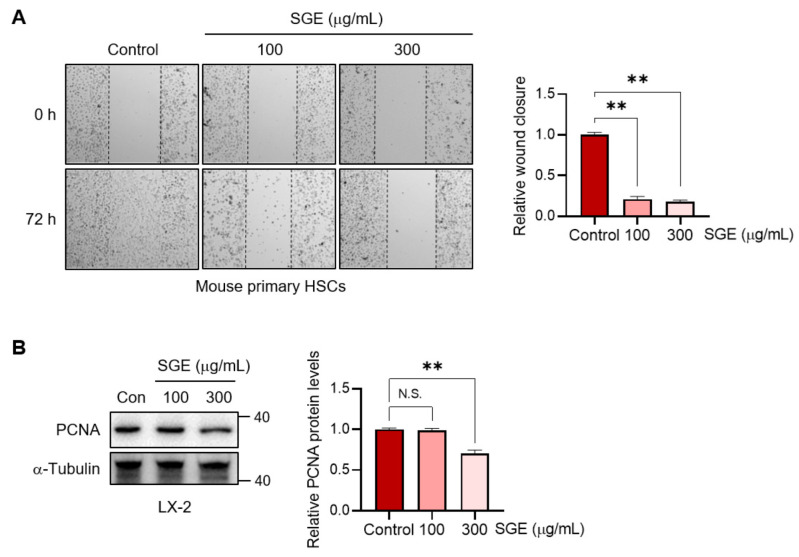
The effects of SGE on the migration and proliferation of HSCs. (**A**) Migration assay in mouse primary HSCs. The cells were treated with SGE (100 or 300 μg/mL) for 72 h. The relative wound closure was quantified (*n* = 3). (**B**) Immunoblotting for PCNA in LX-2 cells (left). The cells were treated with SGE for 24 h. The relative protein levels of PCNA were quantified and normalized to those of α-Tubulin (right) (*n* = 3). The data are presented as mean ± SEM. The statistical significance of the differences between the groups was determined as follows: ** *p* < 0.01 compared with the control group. N.S., not significant.

The activation of HSCs exhibits several features, including migration, proliferation, and contractility, in addition to fibrogenesis [4]. To reinforce our findings, we further examined the effects of SGE treatment on the phenotypes of HSCs. Consistent with the observed decrease in fibrogenic properties, SGE treatment effectively inhibited the migratory abilities of HSCs (Figure 4A). In addition, SGE supplementation slightly but significantly reduced the proliferation of HSCs, as shown by the expression of PCNA (Figure 4B). These findings provide direct evidence that SGE treatment inhibits the activation of HSCs in terms of fibrogenesis, migration, and proliferation, thereby alleviating liver fibrogenesis.

### 3.4. SGE Treatment Blocks TGFβ1-Induced Smad2/3 Signaling in HSCs

**Figure 5:**
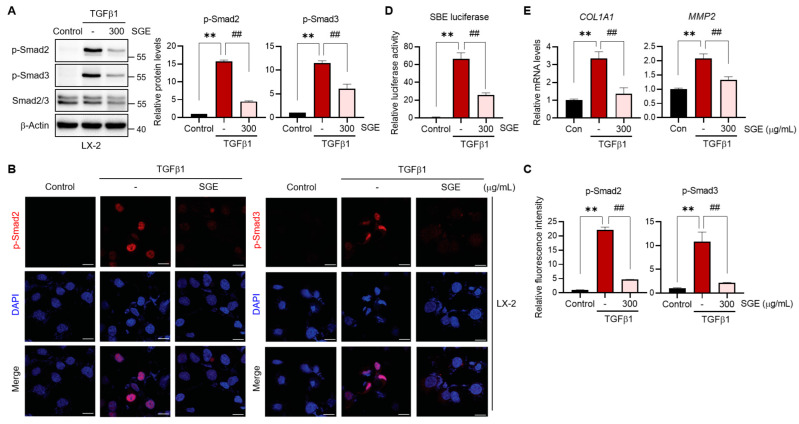
The effect of SGE on TGFβ1-Smad2/3 signaling in HSCs. (**A**) Immunoblotting for p-Smad2/3. LX-2 cells were pre-treated with 300 μg/mL of SGE for 30 min and then exposed to TGFβ1 (5 ng/mL) for 15 min. The relative protein levels of p-Smad2 and p-Smad3 were quantified and normalized to those of β-Actin (*n* = 3). (**B**) Immunofluorescence staining for p-Smad2 and p-Smad3 in LX-2 cells (scale bar: 20 μm). (**C**) Fluorescence intensities of p-Smad2 and p-Smad3 in panel B were quantified (*n* = 3). (**D**) SBE luciferase assay in LX-2 cells. The cells were transfected with the reporter vector and then treated with SGE (300 μg/mL) and TGFβ1 (5 ng/mL, 24 h). (**E**) qRT-PCR assay for fibrogenic genes in LX-2 cells. Like panel D, the cells were similarly treated with SGE (300 μg/mL, 30 min) and TGFβ1 (5 ng/mL, 12 h). The data are presented as mean ± SEM. The statistical significance of the differences between the groups was determined as follows: ** *p* < 0.01 compared with the control group and ## *p* < 0.01 compared with the TGFβ1 alone group.

TGFβ1 is known as the most potent fibrogenic mediator that promotes HSC activation [12]. To elucidate the underlying mechanism by which SGE inhibits HSC activation and liver fibrosis, we examined its effect on TGFβ1 signaling. Treatment of LX-2 cells, a well-established human HSC line, with TGFβ1 resulted in the increased phosphorylation of Smad2 and Smad3, which are canonical downstream components of the TGFβ1 pathway. However, the TGFβ1-induced phosphorylation of Smad2/3 was significantly blocked by SGE treatment (Figure 5A). When Smad2/3 is phosphorylated, it associates with Smad4 to form a complex that relocates to the nucleus, where it prompts the expression of fibrotic genes [13]. Immunofluorescence staining results demonstrated that SGE treatment significantly reduced the nuclear accumulation of p-Smad2 and p-Smad3 induced by TGFβ1 in LX-2 cells (Figure 5B,C). To strengthen the effect of SGE on the TGFβ1-mediated Smad2/3 signaling pathway, we also performed a luciferase reporter assay. In this system, the luciferase expression was controlled by the Smad binding element (SBE). As expected, TGFβ1 efficiently increased the SBE-driven luciferase activities, which were notably suppressed by SGE treatment in LX-2 cells (Figure 5D), providing compelling evidence for the direct regulatory impact of SGE on the Smad2/3 signaling. As a result, the transcript levels of downstream fibrogenic genes (i.e., *COL1A1* and matrix metalloproteinase-2 (*MMP2*)) that were induced by TGFβ1 exhibited efficient attenuation upon supplementation with SGE (Figure 5E). Furthermore, we examined the effects of SGE on non-canonical TGFβ1 pathways and observed that JNK and ERK signaling pathways downstream of TGFβ1 remained unaffected by SGE treatment in LX-2 cells (Figure S3). Overall, these findings support the notion that SGE treatment inhibits HSC activation mainly by blocking TGFβ1-Smad2/3 signaling.

In our search for the functional constituents responsible for the anti-fibrotic effect of SGE, we assessed the effect of quercetin on TGFβ1 signaling in HSCs. Quercetin is a potential compound found in *S. glauca* [14] and has been suggested to possess anti-fibrotic properties [15,16]. Consistent with the effects of SGE, treatment with quercetin significantly inhibited the phosphorylation of Smad2 and Smad3 in LX-2 cells upon TGFβ1 stimulation (Figure S4). Hence, these data suggest that quercetin may be one of the potential anti-fibrotic phytoconstituents of SGE. Nonetheless, further clarification in future studies is warranted.

## 4. Discussion

In the present study, we newly discovered the anti-fibrotic effects of SGE on HSC activation and hepatic fibrogenesis. The administration of SGE effectively alleviated liver fibrosis induced by CCl_4_ in mice, as demonstrated by histological changes, reduced collagen deposition, decreased expression of HSC activation markers, and improvements in blood biochemical parameters. Moreover, SGE treatment efficaciously inhibited early fibrotic and inflammatory responses triggered by acute liver injury caused by a single CCl_4_ injection, providing further support for the inhibitory effects of SGE on the fibrogenesis process. Thus, SGE treatment may offer potential therapeutic resources to cure liver fibrosis. Nevertheless, the effects of SGE on additional diet-induced or cholestatic fibrosis models need to be validated through future studies. 

The activation of HSCs plays a pivotal role in the accumulation of ECM and the development of liver fibrosis [5,11]. In this study, we have provided clear evidence demonstrating that SGE treatment effectively prevents HSC activation. This observation was supported by multiple experiments, including immunoblot analysis, immunocytochemistry, and qRT-PCR. Importantly, the inhibitory effects of SGE were observed in both primary murine HSCs and a human HSC line. A series of our experiments revealed that SGE has the capability to not only suppress HSC fibrogenesis but also to inhibit the migration and proliferation of HSCs. In addition to its impact on hepatic fibrogenesis, HSC activation is also implicated in the induction of hepatocyte injury and the activation of various immune cells within the liver, such as macrophages, neutrophils, and lymphocytes [17]. Our results, which demonstrate an improvement in blood biochemical parameters due to SGE treatment in chronic liver injury but not in acute liver injury, support the idea that the anti-fibrotic effect of SGE treatment may likely be to target HSC activation during fibrogenesis. Therefore, the inhibition of HSC activation by SGE administration may influence the intercellular communication mediated by activated HSCs, which is accountable for the progression of liver fibrosis. 

Communication between HSCs and hepatocytes, the parenchymal cell type of the liver, plays a critical role in the development of hepatic fibrosis. Activated HSCs can contribute to hepatocyte dysfunction and cell death [18,19], while injured hepatocytes can induce HSC activation by altering their secretory patterns, leading to an increase in pro-fibrotic factors and a decrease in anti-fibrotic factors [4,10,20]. It has been suggested that a component of SGE exhibits a protective effect on hepatocytes [9]; therefore, the potential actions of SGE supplementation on both hepatocytes and HSCs may contribute to its anti-fibrotic effects against chronic liver injury in mice, enhancing the therapeutic value of SGE. However, considering that SGE did not induce any changes in the viability of the hepatocyte cell line when challenged with CCl_4_ under our experimental conditions, any potential impact of SGE on hepatocytes needs to be carefully interpreted.

TGFβ1 has a key role in HSC activation and the progress of hepatic fibrogenesis [12]. Another significant finding of this study is the inhibition of TGFβ1 signaling in HSCs by SGE treatment. Upon the binding of TGFβ1 to TGFβ receptor II (TβRII), the receptor forms a dimer with TGFβ1 receptor I (TβRI), leading to the phosphorylation of Smad2/3 [21]. Phosphorylated Smad2 and Smad3 then combine with Smad4 and enter the nucleus where they interact with DNA and induce the transcription of target genes [13]. In our study, SGE supplementation markedly suppressed the TGFβ1-induced phosphorylation of Smad2/3 and their nuclear translocation in LX-2 cells, which was reinforced by the inhibition of SBE-driven transcriptional activities. In contrast, SGE did not affect the TGFβ1-mediated JNK and ERK signaling pathways. These results indicate that the anti-fibrotic effects of SGE may be mediated through the inhibition of TGFβ1-Smad2/3 signaling in HSCs. Notably, TGFβ1 not only induces fibrogenic properties in HSCs but also contributes to hepatocyte injury [22]. Thus, the inhibition of TGFβ1 by SGE may have hepatoprotective effects overall.

Despite the potential value of natural products derived from the Suaeda genus to which *S. glauca* belongs [14], there has been limited research on the biological effects and active constituents of *S. glauca*. A significant aspect of our study involved cultivating *S. glauca* within a smart farming system using LED lamps under tightly controlled conditions. This cultivation method offered several advantages, including standardized plant extract with consistent bioactivity and a reduced risk of heavy metal contamination. Previous reports have suggested that *S. glauca* may contain various substances such as flavonoids and alkaloids [14]. Among these, quercetin has been reported to exhibit beneficial effects on liver fibrosis [15,16]. In the present study, we confirmed that quercetin, like SGE, inhibited the TGFβ1-induced phosphorylation of Smad2/3 in HSCs. Given the importance of reactive oxygen species in HSC activation and inflammatory liver injury [23,24,25], it is worth considering the antioxidant properties of Suaeglaucin B, a compound found in *S. glauca* [8,14]. Therefore, quercetin and Suaeglaucin B might be the putative constituents of interest related to the anti-fibrotic effect of SGE. However, further studies are needed to identify exact anti-fibrotic ingredients and to explore the specific roles of these individual components present in SGE. The validation of the therapeutic effects of SGE will also be required in human subjects.

## 5. Conclusions

Our findings indicate that administering SGE effectively mitigates HSC activation and the progression of hepatic fibrosis in mice. The favorable effects of SGE in inhibiting HSC activation may be associated with the suppression of TGFβ1-induced Smad2/3 signaling in HSCs. However, additional studies are needed to clarify the roles of specific components present in SGE and to validate the therapeutic effects of SGE in human subjects.

## Data Availability

The datasets produced and analyzed during the present study are accessible from the corresponding authors upon reasonable request.

## Supplementary Materials

The following supporting information can be downloaded at: https://www.mdpi.com/article/10.3390/nu15173740/s1, Table S1. The primer sequences for qRT-PCR assays. Figure S1. Body weight of mice treated with CCl_4_ in combination with SGE; Figure S2. Effect of SGE on cytotoxicity in AML12 cells; Figure S3. Effect of SGE on JNK and ERK signaling pathways in HSCs; Figure S4. Effect of quercetin on TGFβ1-induced Smad2/3 signaling in HSCs.

## Abbreviations

ALT: alanine aminotransferase; AST: aspartate aminotransferase; α-SMA: alpha-smooth muscle actin; CCl_4_: carbon tetrachloride; ECM: extracellular matrix; ERK: extracellular signal-regulated kinase; GAPDH: glyceraldehyde-3-phosphate dehydrogenase; H&E: hematoxylin and eosin; HSCs: hepatic stellate cells; JNK: c-Jun N-terminal kinase; MMP: matrix metalloproteinase; PCNA: proliferating cell nuclear antigen; SBE: Smad binding element; SGE: *S. glauca* extract; TGFβ1: transforming growth factor-beta 1.
