# Supplementary material for: Suaeda glauca Attenuates Liver Fibrosis in Mice by Inhibiting TGFβ1-Smad2/3 Signaling in Hepatic Stellate Cells
Source: Nutrients. 2023 Aug 26;15(17):3740. doi: 10.3390/nu15173740 (PMC10490352; doi:10.3390/nu15173740)
Supplement: Supplementary file 1 [file nutrients-15-03740-s001.zip › nutrients-2530104-supplementary.pdf]

Table S1. The primer sequences for qRT-PCR assays

Species	Gene names	Pairs	Primer sequences (5′-3′)
Mouse	*Acta2*	Forward	TGCTGACAGAGGCACCACTGAA
		Reverse	CAGTTGTACGTCCAGAGGCATAG
	Tgfb1	Forward	TGATACGCCTGAGTGGCTGTCT
		Reverse	CACAAGAGCAGTGAGCGCTGAA
	Ccl2	Forward	GCTACAAGAGGATCACCAGCAG
		Reverse	GTCTGGACCCATTCCTTCTTGG
	Ccl7	Forward	CAGAAGGATCACCAGTAGTCGG
		Reverse	ATAGCCTCCTCGACCCACTTCT
	Il1b	Forward	TGGACCTTCCAGGATGAGGACA
		Reverse	GTTCATCTCGGAGCCTGTAGTG
	Col1a1	Forward	ATCTCCTGGTGCTGATGG
		Reverse	GCCTCTTTCTCCTCTCTGA
	Col1a2	Forward	TTCTGTGGGTCCTGCTGGGAAA
		Reverse	TTGTCACCTCGGATGCCTTGAG
	Des	Forward	GTGGATGCAGCCACTCTAGC
		Reverse	TTAGCCGCGATGGTCTCATAC
	Mmp9	Forward	GCAGAGGCATACTTGTACCG
		Reverse	TGATGTTATGATGGTCCCACTTG
	Serpine1	Forward	TTCAGCCCTTGCTTGCCTC
		Reverse	ACACTTTTACTCCGAAGTCGGT
	Timp1	Forward	GCAACTCGGACCTGGTCATAA
		Reverse	CGGCCCGTGATGAGAAACT
	Gapdh	Forward	AACGACCCCTTCATTGAC
		Reverse	TCCACGACATACTCAGCAC
Human	*COL1A1*	Forward	GGACACAGAGGTTTCAGTGG
		Reverse	CCAGTAGCACCATCATTTCC
	MMP2	Forward	GTATTTGATGGCATCGCTCA
		Reverse	CATTCCCTGCAAAGAACACA
	GAPDH	Forward	GAAGATGGTGATGGGATTTC
		Reverse	GAAGGTGAAGGTCGGAGTC

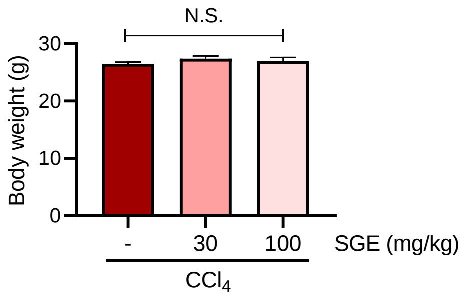

Figure S1. Body weight of mice treated with CCl_4 in combination with SGE

Mice were treated with CCl_4 in the presence or absence of SGE, as shown in Figure 1A (N=6-8 for each group). The data are presented as mean ± SEM. Statistical significance of the differences between the groups was determined as follows: N.S., not significant.

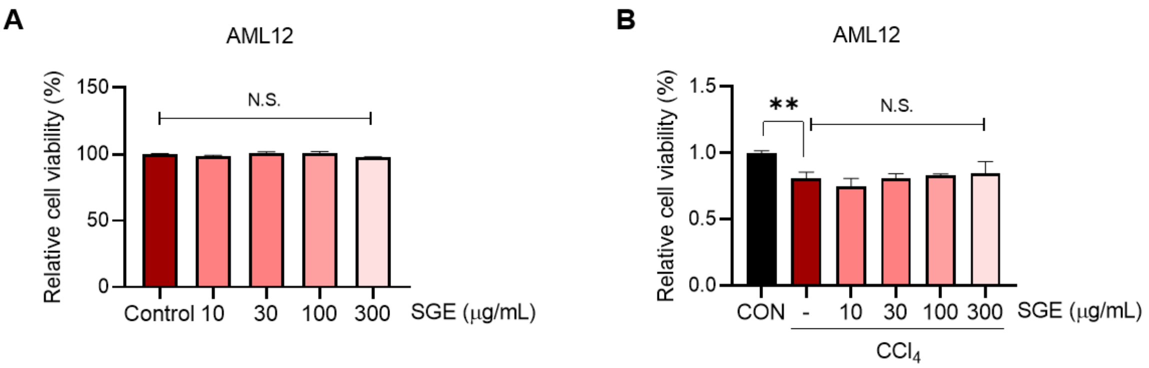

Figure S2. Effect of SGE on cytotoxicity in AML12 cells

(A and B) MTT assay for cell viability in AML12 cells. In panel A, the cells were treated with various concentrations of SGE as indicated for a duration of 24 hours. In panel B, the cells were exposed to CCl_4 (5 mM, 24 h) following pre-treatment with SGE. The data are presented as mean ± SEM. Statistical significance of the differences between the groups was determined as follows: $^{**}p<0.01$ compared to the control group. N.S., not significant.

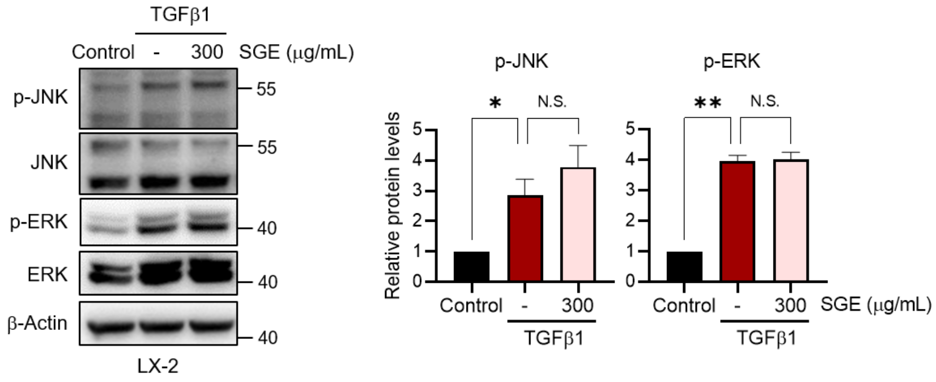

Figure S3. Effect of SGE on JNK and ERK signaling pathways in HSCs

Immunoblotting for p-JNK and p-ERK. LX-2 cells were pre-treated with 300 μg/mL of SGE for 30 min, and then exposed to TGFβ1 (5 ng/mL) for 15 min. The relative protein levels of p-JNK and p-ERK were quantified and normalized to those of β-Actin (N=3). The data are presented as mean ± SEM. Statistical significance of the differences between the groups was determined as follows: *p<0.05 or **p<0.01 compared to the control group. N.S., not significant.

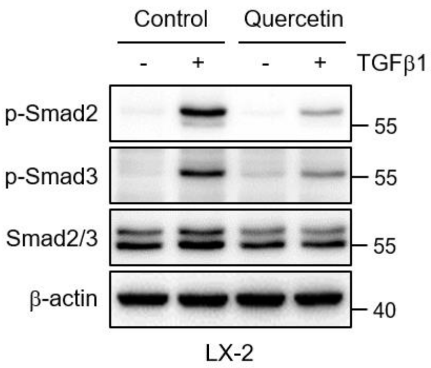

Figure S4. Effect of quercetin on TGFβ1-induced Smad2/3 signaling in HSCs

Immunoblotting for p-Smad2/3. LX-2 cells were treated with quercetin (100 μM, 30 min) in combination with TGFβ1 (5 ng/mL, 15 min).
